# Supplementary material for: Limited and localized magmatism in the Central Atlantic Magmatic Province
Source: Nat Commun. 2020 Jul 7;11:3397. doi: 10.1038/s41467-020-17193-6 (PMC7341742; doi:10.1038/s41467-020-17193-6)
Supplement: Supplementary file 1 — Supplementary Information [file 41467_2020_17193_MOESM1_ESM.pdf]

Supplementary Information

Limited and localized magmatism in the Central Atlantic Magmatic Province

R.E. Marzen[1*], D.J. Shillington[1,6], D. Lizarralde[2], J.H. Knapp[3], D.M. Heffner[4,7], J.K. Davis[1,8], S.H. Harder[5]

[1]Lamont-Doherty Earth Observatory of Columbia University, Palisades, NY 10964
[2]Woods Hole Oceanographic Institution, Woods Hole, MA 02543
[3]Oklahoma State University, Stillwater, OK 74078
[4]University of South Carolina, Columbia, SC 29208
[5]University of Texas El Paso, El Paso, TX 79902
[6]now at Northern Arizona University, Flagstaff, AZ 86011
[7]now at Occidental Petroleum Corporation, Houston, TX 77046
[8]now at Drummond Carpenter, PLLC, Orlando, FL 32801

Contents

Supplementary Table 1. SUGAR Line 1 Shot Information
Supplementary Table 2. Data misfit by shot for Line 1
Supplementary Table 3. Data misfit by phase for Line 1
Supplementary Table 4. Calibration Parameters for High and Low Velocity Estimates of Intrusions

Supplementary Figure 1. Map of the SUGAR Lines 1 and 2 with shots numbered
Supplementary Figure 2. Comparison of southeastern-most shot gathers
Supplementary Figure 3. Example Line 1 shot gathers and raypaths
Supplementary Figure 4. Line 1 pick and ray coverage statistics
Supplementary Figure 5. Line 2 pick and ray coverage statistics
Supplementary Figure 6. Line 1 tradeoff test
Supplementary Figure 7. Line 2 tradeoff test
Supplementary Figure 8. Decompression melting calculations for depth-dependent lithospheric thinning
Supplementary Figure 9. Decompression melting calculations for different initial lithosphere thicknesses
Supplementary Figure 10. Relationship between synrift sediment thickness and estimated magmatic intrusion thickness

Supplementary Note 1. Constraints on basin structure
Supplementary Note 2. Tradeoff tests

Supplementary References

Supplementary Table 1. SUGAR Line 1 Shot Information See Supplementary Fig. 1 for locations.

Shot Number	*Time (YYYY,JDD,hr,mn,ss.sss)*	*Latitude*	*Longitude*	*Charge Size (kg)*
1	2014:079:03:05:00.02	32.68682	-84.98628	635
3	2014:079:03:10:00.02	32.30797	-84.59778	227
4	2014:078:03:00:00.02	32.16044	-84.46409	227
5	2014:078:03:10:00.02	32.02107	-84.36591	227
6	2014:078:04:25:00.02	31.80909	-84.11940	45
7	2014:078:05:11:00.02	31.73172	-84.04466	45
8	2014:078:06:10:00.02	31.57942	-83.85434	91
10	2014:078:07:05:00.02	31.21521	-83.50206	181
11	2014:078:06:05:00.02	31.09683	-83.40052	181
13	2014:078:04:35:00:02	30.80598	-83.12720	204
14	2014:078:03:05:00.02	30.65708	-82.96895	817

Supplementary Table 2. Data misfit by shot for Line 1

Shot	*RMS misfit (s)*	*χ^2 misfit*	*Number of picks*
1	0.06148188	1.08162332	442
3	0.07692094	1.65698028	841
4	0.06913318	1.15784931	879
5	0.0736941	1.14978492	845
6	0.06145826	0.89686519	740
7	0.07368409	1.38962281	768
8	0.0842474	1.53135049	736
10	0.07812853	1.47783065	699
11	0.08328903	1.61050785	290
13	0.09087306	1.92047346	376
14	0.05691192	0.78356344	1078

Supplementary Table 3. Data misfit by phase for Line 1

Phase	*RMS misfit (s)*	*χ^2 misfit*	*Number of picks*
Pg	0.06244588	1.20946491	5334
PmP	0.09152883	1.37546265	1812
Pn	0.09913809	1.5366745	548

Supplementary Table 4. Calibration Parameters for High and Low Velocity Estimates of Intrusions

Bin: Min Thickness (m)	*Bin: Max Thickness (m)*	*SGR Area (m^2) from Heffner Basin Model*	*Average Deep Crustal Mafic Intrusion Thickness (km) - High Estimate 7.2 km/s Intrusion Velocity*	*Average Deep Crustal Mafic Intrusion Thickness (km) - Low Estimate 7.5 km/s Intrusion Velocity*
3500	4500	476707201.5	5.84	3.50
3000	3500	1888456513	5.37	3.22
2500	3000	4020881323	5.30	3.18
2000	2500	7428096363	3.80	2.28
1500	2000	11885821199	2.34	1.40
1000	1500	16818437735	2.20	1.32
500	1000	40581570711	Assume 0	Assume 0
0	500	Not Calculated	Assume 0	Assume 0

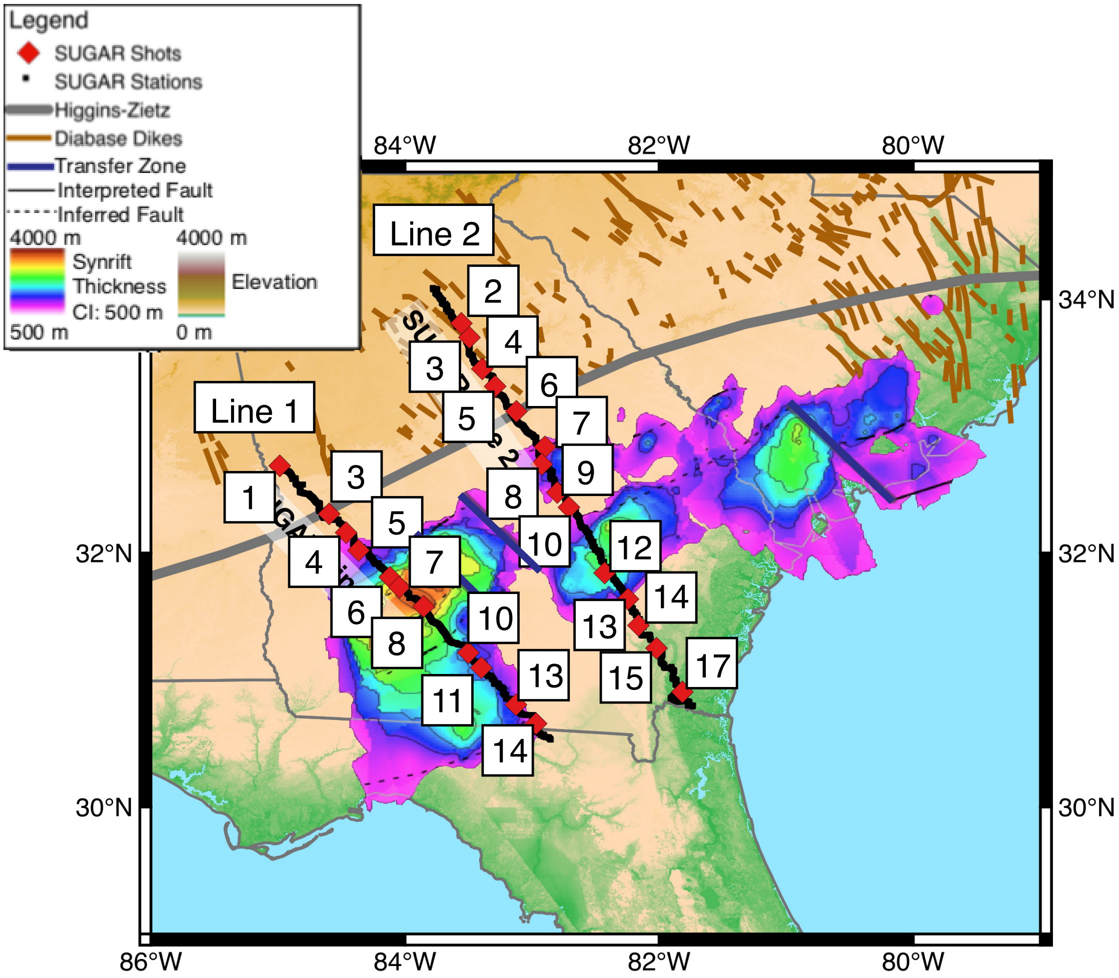

Supplementary Figure 1. Map of the SUGAR Lines 1 and 2 with shots numbered. Synrift sediment thickness from Heffner (2013). Diabase dikes from Ragland et al. (1983) and King (1961).

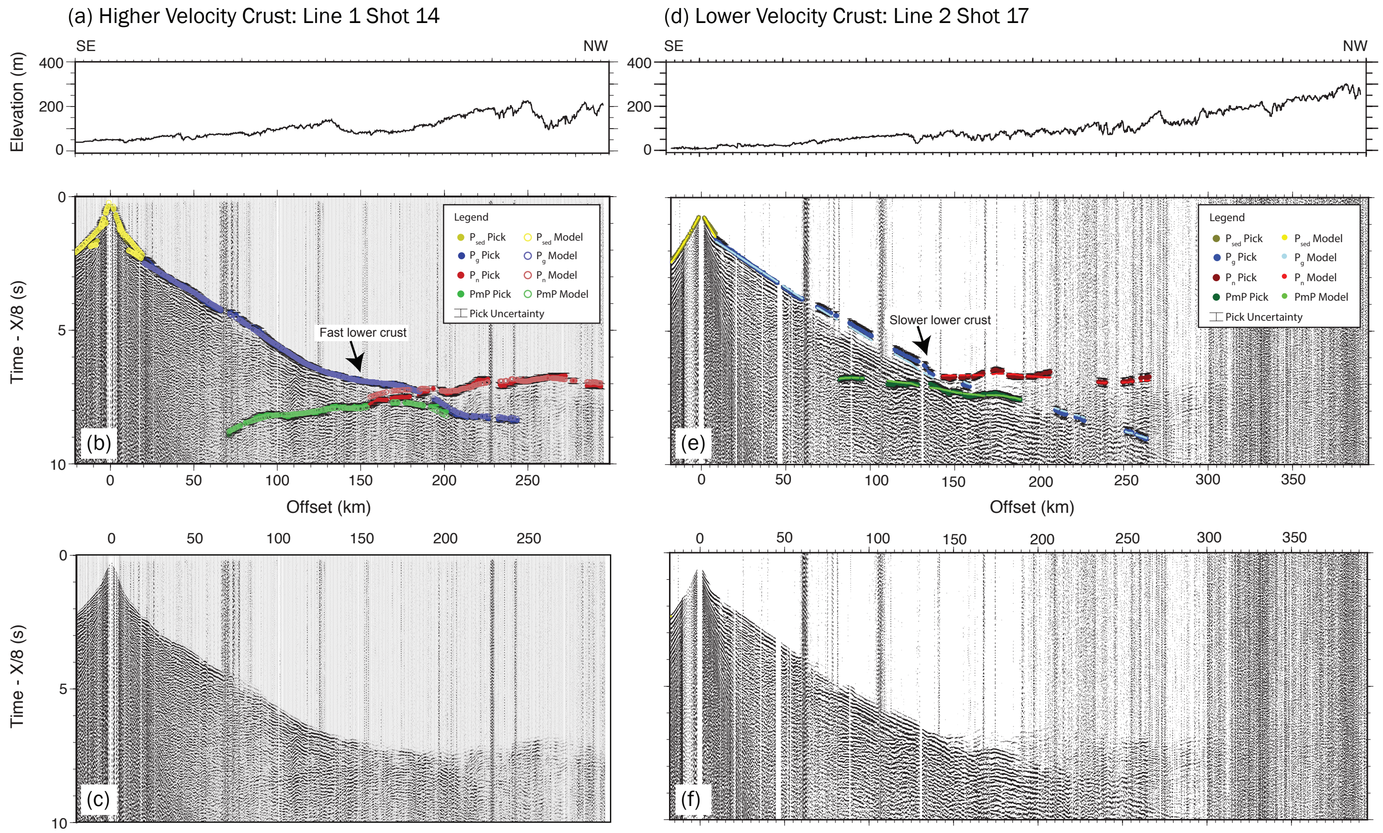

Supplementary Figure 2. Comparison of southeastern-most shot gathers (a-c) SUGAR Line 1 (Shot 14) and (d-f) SUGAR Line 2 (Shot 17; See Supplementary Fig. 1 for locations). (a,d) Elevation along the two profiles. (b,e) Shot gathers plotted at a reduction velocity of 8 km s^{-1} with model predicted picks (light open circles) and observed picks (dark filled circles with bars indicating uncertainty 1σ). (c,f) Uninterpreted shot gathers. The difference in slope of long-offset Pg arrivals between the two lines is indicative of the higher lower crustal velocities observed on Line 1 compared to Line 2.

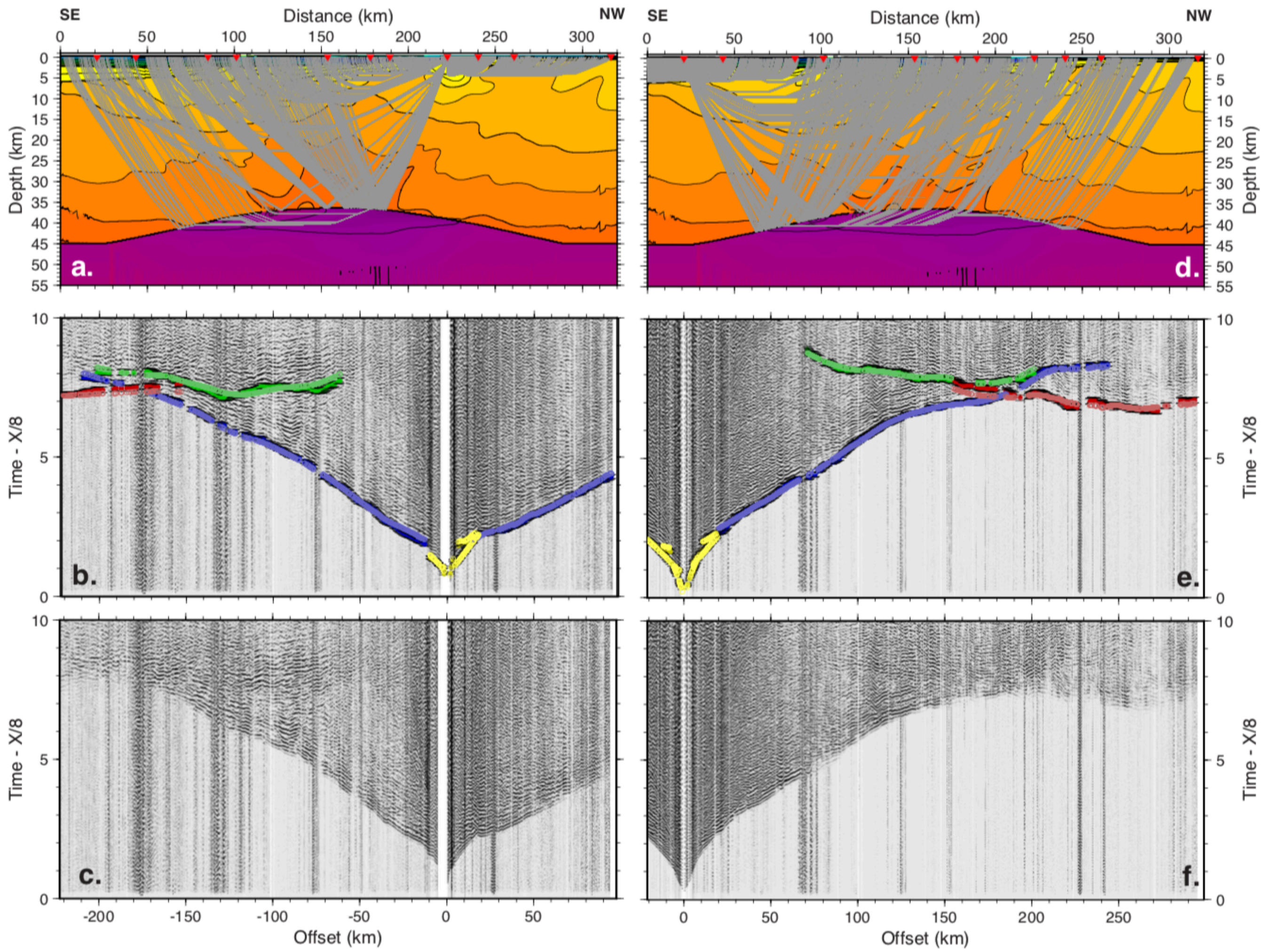

Supplementary Figure 3. Example Line 1 shot gathers and raypaths (a-c) Line 1 Shot 5 and (d-f) Line 1 Shot 14 (see Supplementary Fig. 1 for locations). (a,d) Final velocity models and raypaths through the final velocity model in grey. Shot locations along the line indicated with red inverted triangles. Contour interval at 0.5 km/s. See Fig. 2 in manuscript for color bar. Vertical exaggeration: 2. (b,e) Shot gathers with every 10th predicted (open circles) and observed travel time pick (filled circles with uncertainty bars). (c,f) Plot of shot gathers without interpreted arrivals.

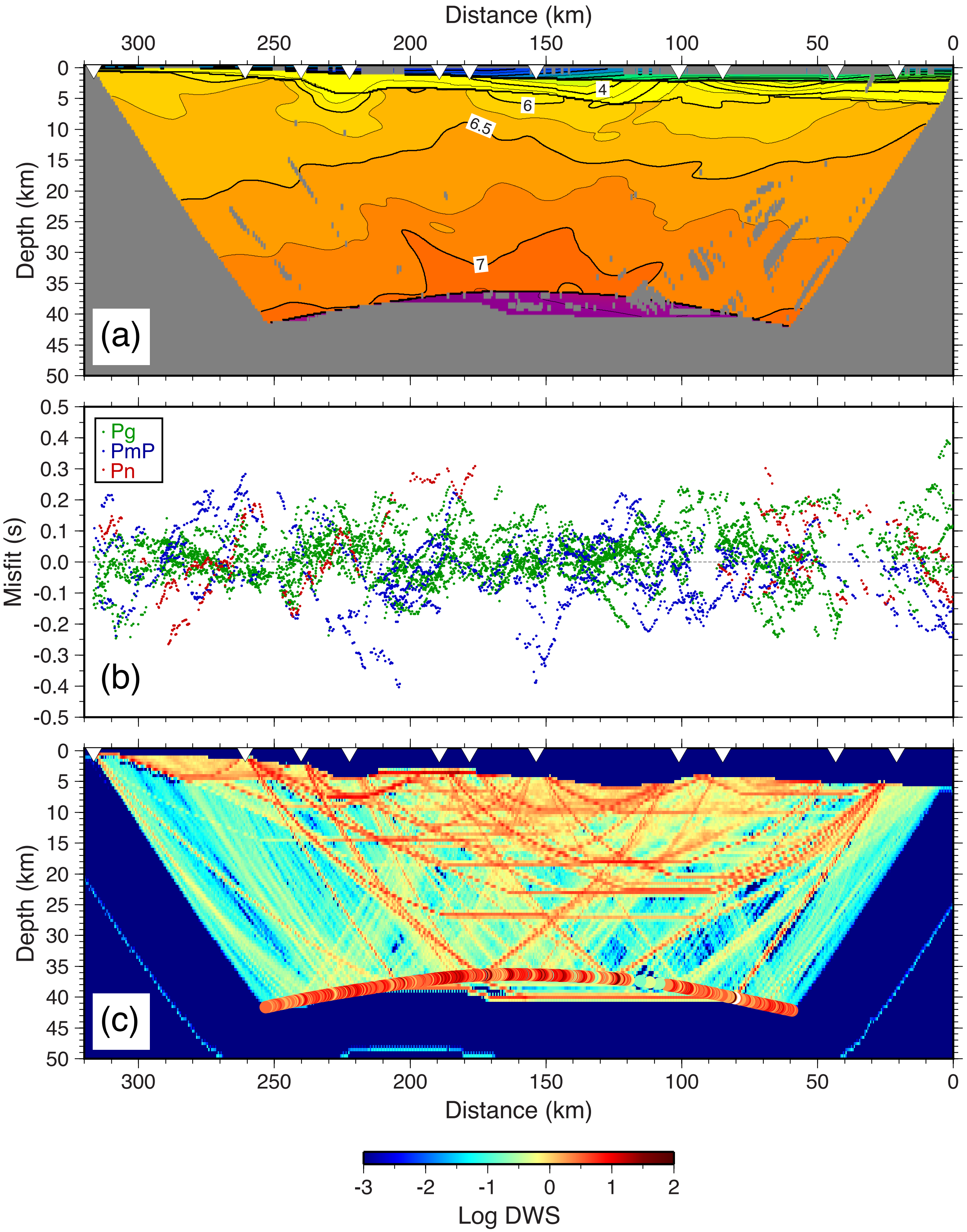

Supplementary Figure 4. Line 1 pick and ray coverage statistics (a) Line 1 velocity model masked by ray coverage. (b) Plot of pick misfit colored by arrival type. (c) Plot of the log of the derivative weight sum statistic for the velocity model (grid color) and Moho (colored circles) as an indicator of resolution.

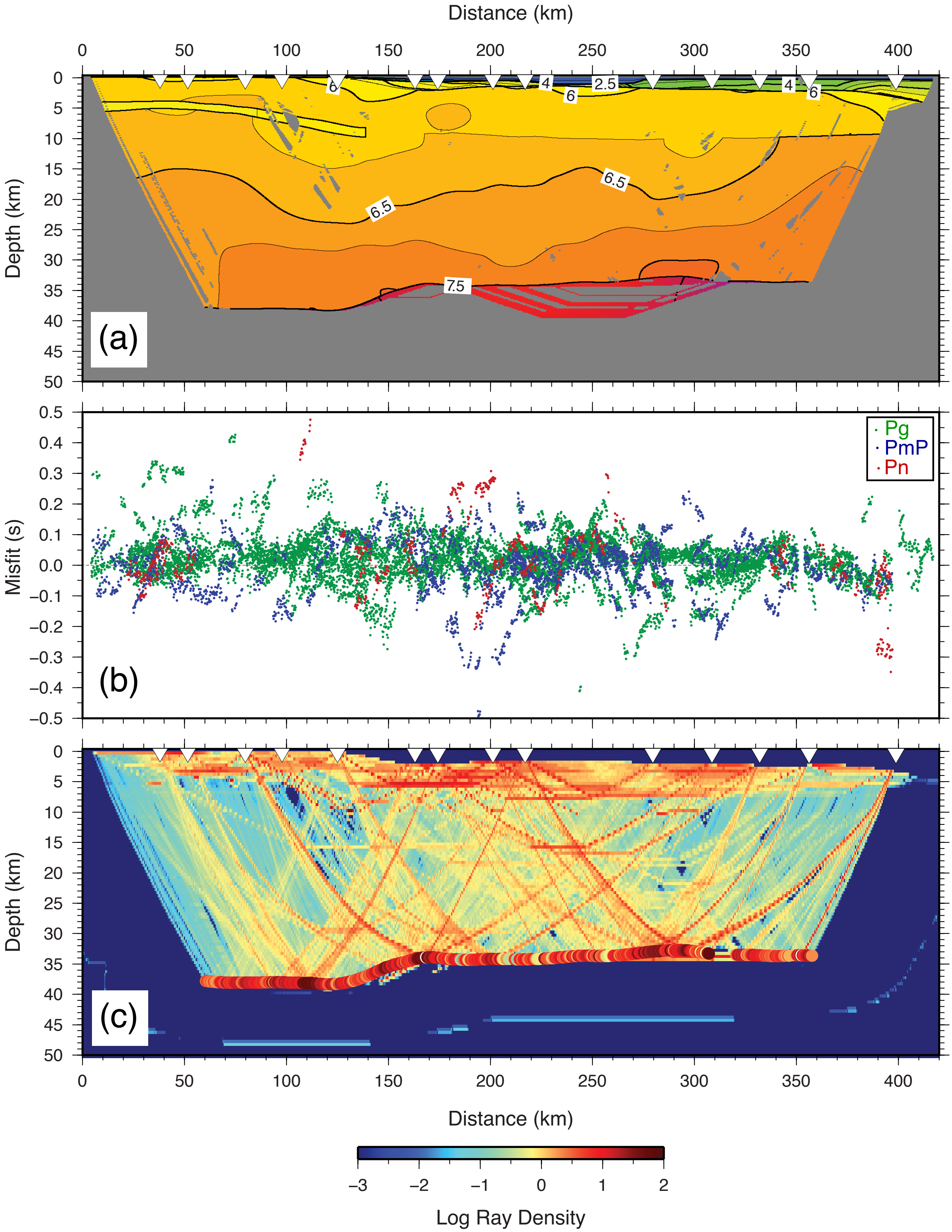

Supplementary Figure 5. Line 2 pick and ray coverage statistics (a) Line 2 velocity model masked by ray coverage. (b) Plot of pick misfit colored by arrival type. (c) Plot of the log of the derivative weight sum statistic for the velocity model (grid color) and Moho (colored circles) as an indicator of resolution.

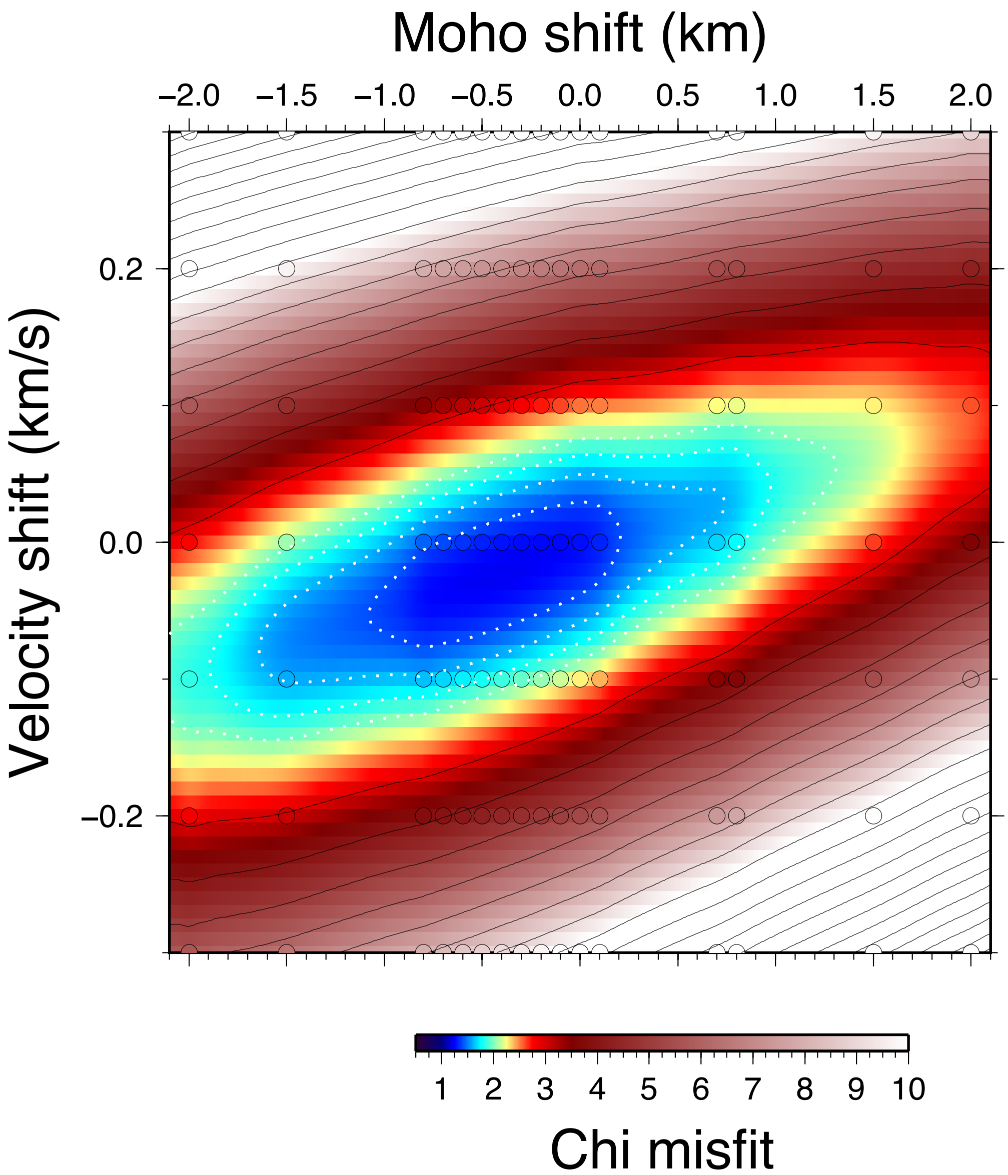

Supplementary Figure 6. Line 1 tradeoff test Gridded chi-squared misfit of long-offset Pg, Pmp and Pn for velocity models of SUGAR Line 1 as a function of perturbations in lower crustal velocity and Moho depth from the final model (Fig. 2). The contour intervals are 1 above chi-squared misfit of 2 (black lines) and 0.2 below 2 (white dotted lines). The innermost white contour is 1.4. Black circles show misfit values that went into gridding. This test shows that models with a slightly thinner crust (~0.75 km) and slower velocity (-0.05 km/s) than our final model could satisfy the travel time data.

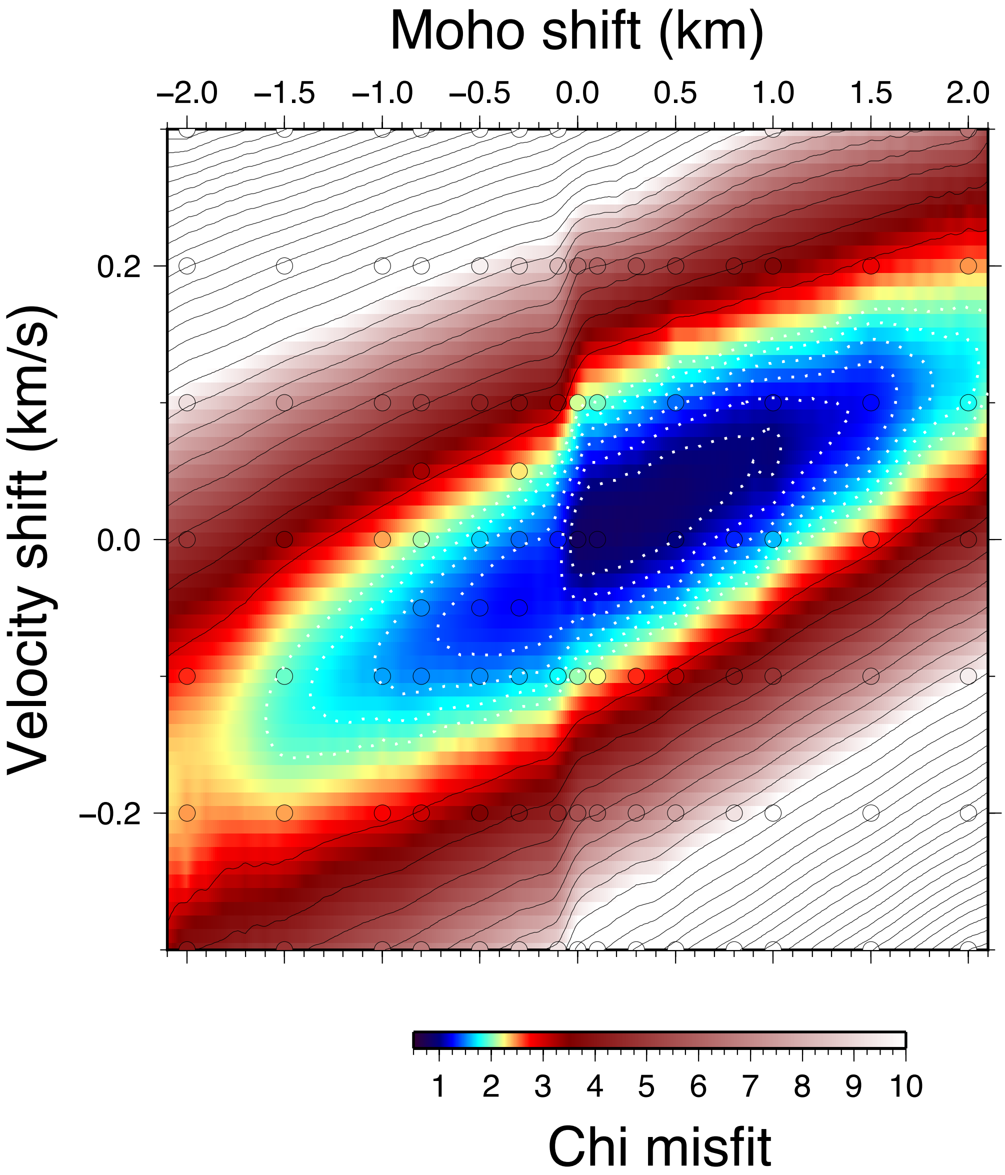

Supplementary Figure 7. Line 2 tradeoff test Gridded chi-squared misfit of lower crustal Pg, Pmp and Pn for velocity models of SUGAR Line 2 as a function of perturbations in lower crustal velocity and Moho depth from the final model (Fig. 2). The contour intervals are 1 above chi-squared misfit of 2 (black lines) and 0.2 below 2 (white dotted lines). The innermost white contour is 1.0. Black circles show misfit values that went into gridding. This test shows that models with a slightly thicker crust (~0.75 km) and higher velocity (+0.05 km/s) than our final model could satisfy the travel time data.

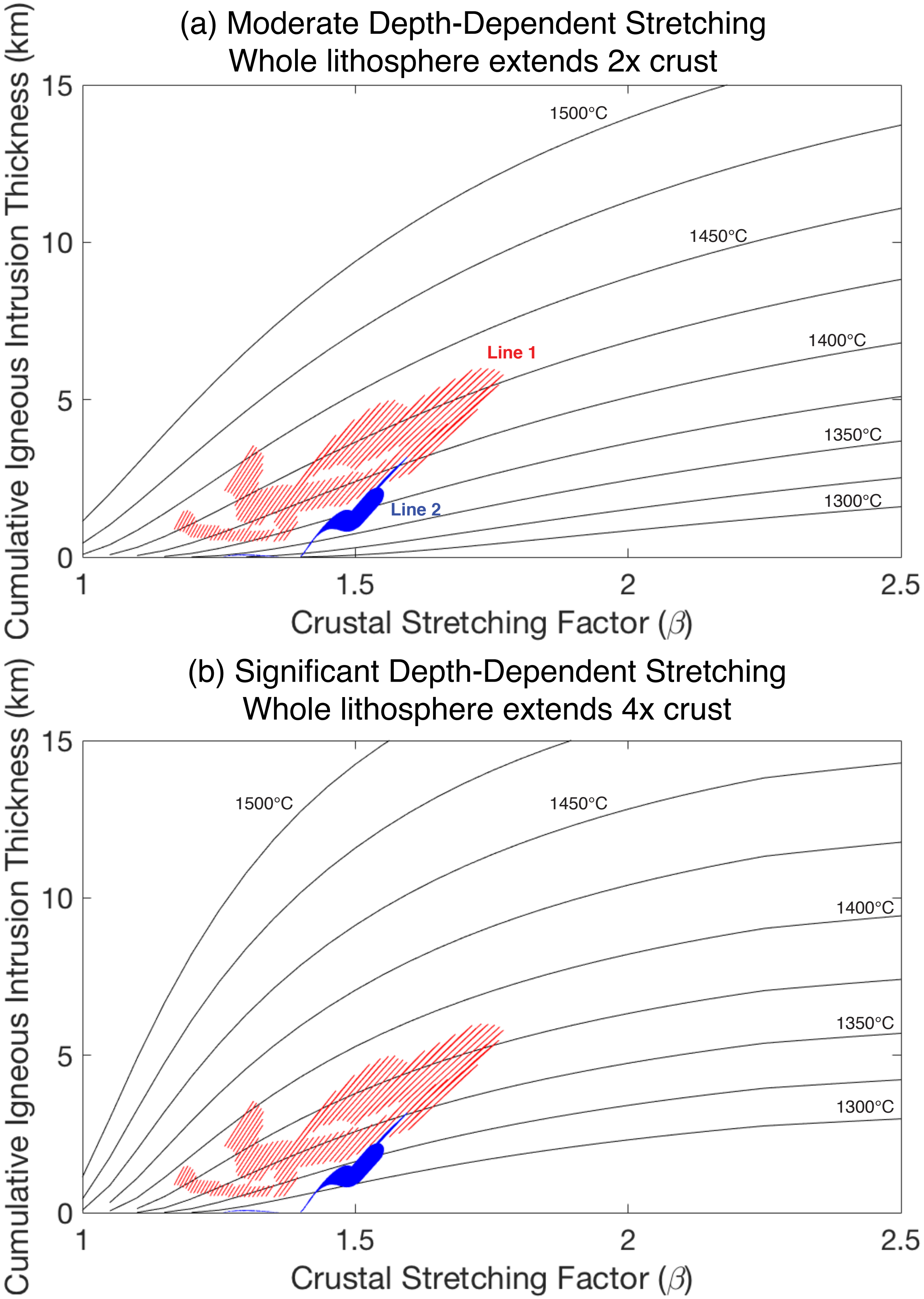

Supplementary Figure 8. Decompression melting calculations for depth-dependent lithospheric thinning (a) Moderate depth-dependent stretching where the whole lithosphere extends 2x as much as the crust. (b) Significant depth-dependent stretching where the whole lithosphere extends 4x as much as the crust.

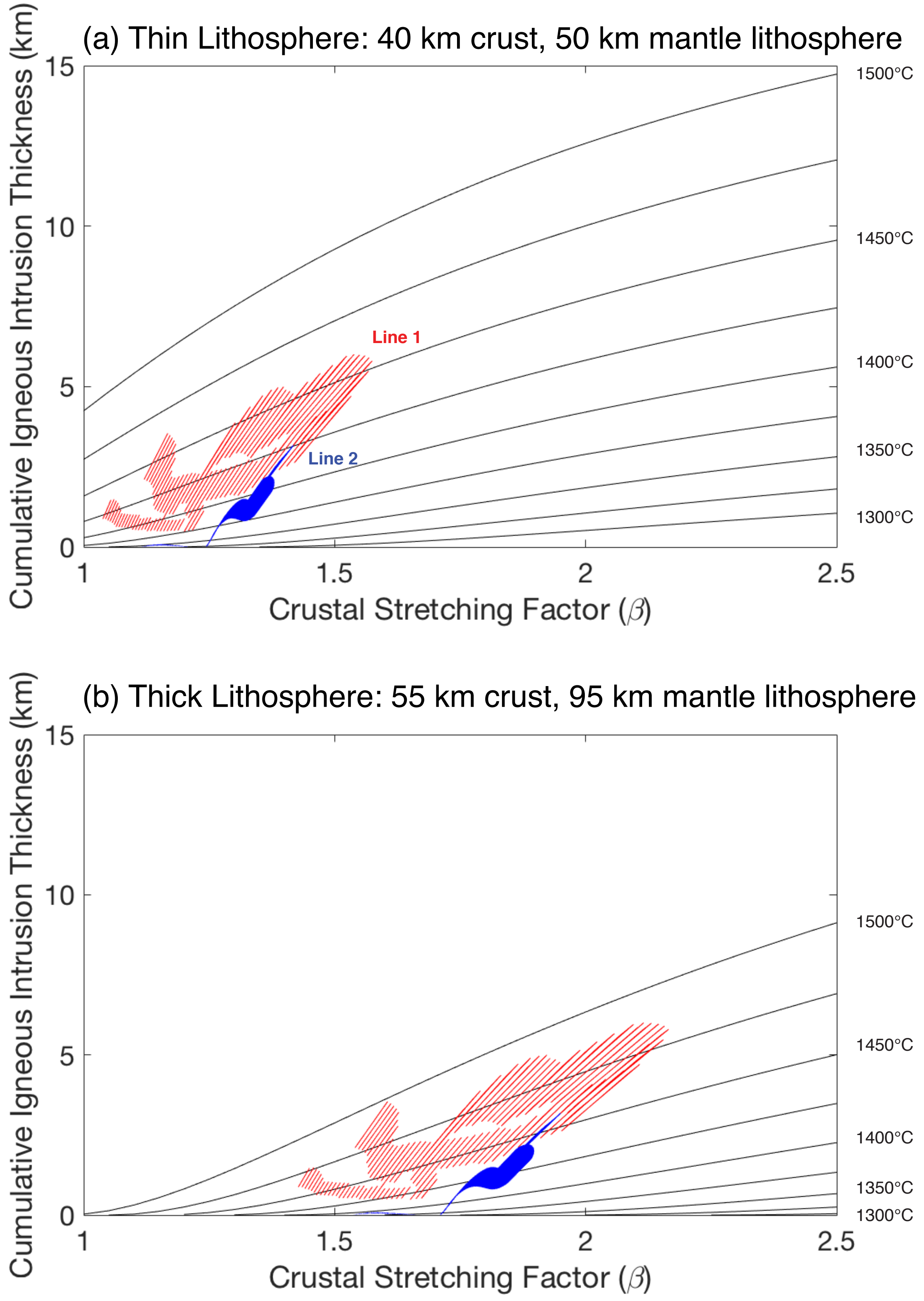

Supplementary Figure 9. Decompression melting calculations for different initial lithosphere thicknesses (a) The thin lithosphere scenario has a 40 km crust, 50 km mantle lithosphere and (b) the thick lithosphere scenario has a 55 km crust, 95 km mantle lithosphere.

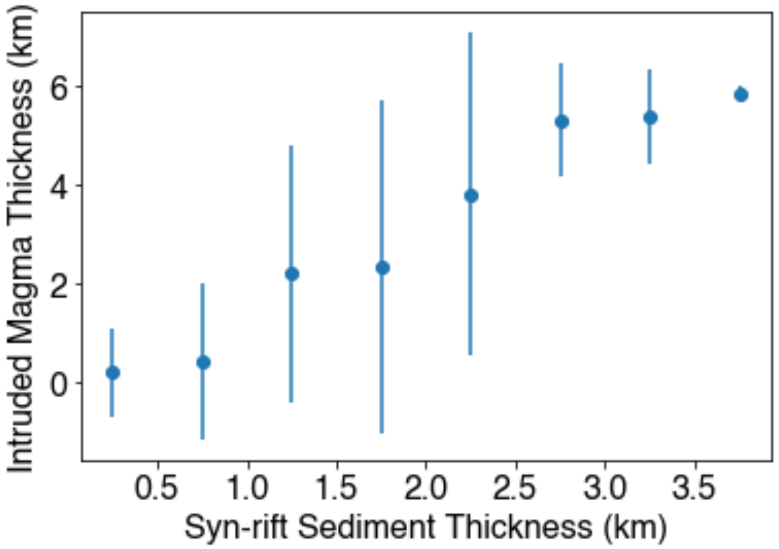

Supplementary Figure 10. Relationship between synrift sediment thickness and estimated magmatic intrusion thickness This plot assumes an intrusion velocity of 7.2 km s^{-1}. Error bars show the 2σ (95%) range in magma intrusion thickness for each 500m bin of syn-rift sediment thickness.

Supplementary Note 1. Constraints on basin structure

A detailed description of the constraints and methods behind the South Georgia Basin model used in this manuscript is published in Chapter 3 of Heffner (2013) and briefly summarized here. This is the first isopach of sedimentary fill in the South Georgia Basin developed by integrating seismic reflection data (Domoracki, 1995; Petersen et al., 1984; Cook et al., 1981; Nelson et al., 1985; Behrendt, 1985; McBride, 1991; Akintunde et al., 2013; Hamilton et al., 1983; Schilt et al., 1983; Yantis et al., 1983; Chapman and Beale, 2010; Behrendt et al., 1983), seismic refraction data (Bonini and Woollard, 1960; Ackermann, 1983; Amick, 1979; Pooley, 1960; Smith, 1982; Woollard, 1957, Cook et al., 1981), and well data (Falls and Prowell, 2001; Applin, 1951; SCDNR; Cumbest et al., 1992; Aadland et al., 1995; Barnett et al., 1975; Snipes et al., 1995; Costain et al., 1986; Marine and Siple, 1974; Steele and Colquhoun, 1985; Ball et al., 1988; Gohn et al., 1983; Gohn, 1983; Scholle, 1979; Chowns and Williams, 1983; Falls, 1994; Herrick, 1961; Applin and Applin, 1964; McFadden et al., 1986; Milton and Hurst, 1965; Neathery and Thomas, 1975; Gellici, 2007; Mansfield, 1937; Dillon and Popenoe, 1988; additional log data and core observations) throughout the Coastal Plain of the southeastern U.S. Regional 2-D deep seismic reflection profiles from COCORP and SEISDATA were of greatest value in constraining sedimentary basin fill thickness based on subsurface stratal geometries. In some cases, interpretations of these geometries were compromised by the presence of high-impedance mafic sills within the basin stratigraphy. Legacy petroleum exploration wells were instrumental in (1) substantiating the presence of continental redbeds of inferred Triassic age beneath the Coastal Plain unconformity, and (2) in limited cases, constraining the base of these deposits for thickness estimates. The thickness of the deeper, relatively high velocity (~4-5.5 km/s) sedimentary layer from the SUGAR velocity models corresponds to first order with the thickness of Heffner's South Geogia Basin isopach model. The two models diverge, though, to the southeast. The basin isopach model shows the South Georgia Basin thinning to the SE, but the fast layer of SUGAR sediments remains thick to the southeastern end of both seismic lines. These fast sediments to the SE on both seismic lines were likely deposited after South Georgia rifting, but may be older and more compacted than coastal plain sediments, resulting in higher seismic velocities.

There are pros and cons to both datasets. The isopach model has a fair amount of well control to constrain where the red beds are located, but limited wells that are deep enough to hit basement and constrain basin thickness. Available reflection seismic data are an important constraint on basin geometry in the isopach model, but seismic reflection lines are limited, and assumptions are made about basin velocities to convert basin thickness in two-way travel time to depth. Imaging of the basement is also sometimes difficult below sills in the basin. While the SUGAR seismic data do constrain basin velocities, basin geometry is largely inferred through indirect constraints between shots based on topography on Pg arrivals. The inability to distinguish between pre-rift and post-rift sediments based on velocity contrasts becomes problematic at the southeastern ends of both SUGAR transects.

Given the fact that SUGAR constraints are limited to individual seismic lines, the first-order agreement on syn-rift sediment thickness between the South Georgia Basin isopach model and the SUGAR seismic data supports the validity of using the isopach model to constrain magma intrusion thickness beneath the South Georgia Rift.

Supplementary Note 2: Tradeoff tests

One of the primary new observations presented in this study is that the crust beneath the western part of the South Georgia Rift, which has thicker synrift sediments, also has higher velocities in the lower crust, which we interpret as mafic magmatic intrusions. However, there can be tradeoffs between lower crustal velocity and Moho depth in refraction studies because of limited refractions through the lower crust. To examine possible tradeoffs in our model, we systematically perturbed the lower crustal velocity and Moho depth from our final

model, traced rays through the perturbed models and calculated the misfits. Moho depths were varied by +/- 2 km at intervals between 0.1 and 0.5 km. We perturbed lower crustal velocities below 27 km, the approximate depth where velocities > 7 km/s are observed, by +/- 0.8 km/s at intervals of 0.05 to 0.1 km/s. To avoid the introduction of a sharp change in velocity with depth in these perturbed models, a gradual change in velocity was applied over a 5-km window between depths of 22 and 27 km, where the velocity adjustment is 0 km/s at 22 km and linearly increased to the full perturbation at 27 km. The misfit was calculated using crustal refractions observed at offsets greater than 120 km (which sampled the middle and lower crust), reflections from the Moho and upper mantle refractions. The latter were included because the crossover of Pg and Pn is sensitive to lower crustal velocity and crustal thickness. Supplementary Figs. 6 and 7 show χ^2 misfit as a function of lower crustal velocity and Moho depth perturbations for Lines 1 and 2. In both, a clear minimum is observed for small perturbations from our final model. As expected, this misfit function does illuminate a small trade-off, where models with deeper Moho and higher velocities or shallower Moho and lower velocities could satisfy our results. On Line 1, if we would allow for χ^2 values to increase from 1.27 (misfit with 0 perturbation) to as high as 1.6, velocities could be as much as 0.05 km/s lower than observed. Similarly, on Line 2, if we would allow χ^2 values to increase from 0.82 (misfit with 0 perturbation) to 1.0, velocities could be as much as ~0.05 km/s higher, SUGAR Line 1 models without elevated lower crustal velocities, which would be comparable to SUGAR Line 2, cannot explain observed travel time arrivals. We owe these relatively tight constraints on lower crustal velocity to long-offset crustal refractions observed in our dataset (e.g., Supplementary Fig. 2).

Supplementary References

1. Aadland, R.K., Gellici, J.A., and Thayer, P.A., 1995, Hydrogeologic framework of west- central South Carolina: South Carolina Department of Natural Resources Water Resources Division Report 5, 200 p.
2. Applin, P.L., 1951, Preliminary report on buried pre-Mesozoic rocks in Florida and adjacent states: US Geological Survey Circular 91, 28 p.
3. Applin, E.R., and Applin, P.L., 1964, Logs of selected wells in the Coastal Plains of Georgia: Georgia Geologic Survey Bulletin 74, 229 p.
4. Ackermann, H.D., 1983, Seismic-refraction study in the area of the Charleston, South Carolina, 1886 earthquake, *in* Gohn, G.S., ed., Studies Related to the Charleston, South Carolina, Earthquake of 1886—Tectonics and Seismicity: U.S. Geological Survey Professional Paper 1313, p. F1–F20.
5. Akintunde, O.M., Knapp, C.C., Knapp, J.H., and Heffner, D.M., 2013, New constraints on buried Triassic basins and regional implications for subsurface CO2 storage from the SeisData6 seismic profile across the Southeast Georgia Coastal Plain: Environmental Geosciences, v. 20, p. 1-13.
6. Amick, D.C., 1979, Crustal structure studies in the South Carolina Coastal Plain, M.S. thesis, University of South Carolina, 81 p.
7. Ball, M.M., Martin, R.G., Foote, R.Q., and Applegate, A.V., 1988, Structure and stratigraphy of the Western Florida Shelf, Part I, Multichannel reflection seismic data: US Geological Survey Open File Report 88-439.
8. Barnett, R.S., 1975, Basement structure of Florida and its tectonic implications: Transactions of the Gulf Coast Association of Geological Societies, v. 25, p. 122- 140.
9. Behrendt, J.C., 1985, Interpretations from multichannel seismic-reflection profiles of the deep crust crossing South Carolina and Georgia from the Appalachian mountains to the Atlantic coast: U.S. Geological Survey U.S. Misc. Field Studies, Map MF- 1656.
10. Behrendt, J.C., Hamilton, R.M., Ackermann, H.D., Henry, V.J., and Bayer, K.C., 1983, Marine multichannel seismic-reflection evidence for Cenozoic faulting and deep crustal structure near Charleston, South Carolina, *in* Gohn, G.S., ed., Studies Related to the Charleston, South Carolina, Earthquake of 1886—Tectonics and Seismicity: U.S. Geological Survey Professional Paper 1313, p. J1–J29.
11. Bonini, W.E., and Woollard, G.P., 1960, Subsurface geology of North Carolina – South Carolina Coastal Plain from seismic data: Bulletin of the American Association of Petroleum Geologists, v. 44, p. 298-315.
12. Chapman, M.C., and Beale, J.N., 2010, On the Geologic Structure at the Epicenter of the 1886 Charleston, South Carolina, Earthquake: Bulletin of the Seismological Society of America, v. 100, p. 1010–1030, doi:10.1785/0120090231.

13. Chowns, T.M., and Williams, C.T., 1983, Pre-Cretaceous rocks beneath the Georgia Coastal Plain—Regional Implications, *in* Gohn, G.S., ed., Studies Related to the Charleston, South Carolina, Earthquake of 1886—Tectonics and Seismicity: U.S. Geological Survey Professional Paper 1313, p. L1–L42.
14. Cook, F.A., Brown, L.D., Kaufman, S., Oliver, J.E., Petersen, T.A., 1981, COCORP seismic profiling of the Appalachian orogen beneath the Coastal Plain of Georgia: Geological Society of America Bulletin, Part I, v. 92, p. 738 – 748.
15. Costain, J.K, Speer, J.A., Glover III, L., Perry, L., Dashevsky, S., and McKinney, M. (1986), Heat flow in the Piedmont and Atlantic Coastal Plain of the Southeastern United States: Journal of Geophysical Research, v. 91, p. 2123-2135.
16. Cumbest, R.J., Price, V., and Anderson, E.E., 1992, Gravity and magnetic modeling of the Dunbarton Triassic basin, South Carolina: Southeastern Geology, v. 33, p. 37- 51.
17. Dillon, W.P., and Popenoe, P., 1988, The Blake Plateau basin and Carolina Trough, *in* Sheridan, R.E., and Grow, J.A., eds., The Atlantic Continental Margin, U.S.: Geological Society of America, Geology of North America, I-2, p. 291-328.
18. Domoracki, W.J., 1995, A Geophysical investigation of geologic structure and regional tectonic setting at the Savannah River Site, South Carolina, PhD thesis, Virginia Polytechnic Institute & State University, Blacksburg, 236 p.
19. Falls, W.F., and Prowell, D.C., 2001, Stratigraphy and depositional environments of sediments from five cores from Screven and Burke counties, Georgia: United States Geological Survey Professional Paper 1603-A, 22 p.
20. Falls, W.F., 1994, Lithologic descriptions of two cores and ground-water-quality data from five counties in the northeastern part of the Coastal Plain of South Carolina, 1988 and 1991: United States Geological Survey Open-File Report 94-58, 49 p.
21. Gellici, J.A., 2007, Hydrostratigraphy of the ORG-393 core hole at Orangeburg, South Carolina: South Carolina Department of Natural Resources Water Resources Report 42, 40 p.
22. Gohn, G.S., 1983, Geology of the basement rocks near Charleston, South Carolina—Data from detrital rock fragments in lower Mesozoic(?) rocks, *in* Clubhouse Crossroads test hole #3, in Gohn, G.S., ed., Studies Related to the Charleston, South Carolina, Earthquake of 1886—Tectonics and Seismicity: U.S. Geological Survey Professional Paper 1313, p. E1–E22.
23. Gohn, G.S., Houser, B.B., and Schneider, R.R., 1983, Geology of the Lower Mesozoic(?) sedimentary rocks in Clubhouse Crossroads Test Hole #3, near Charleston, South Carolina, in Gohn, G.S., ed., Studies Related to the Charleston, South Carolina, Earthquake of 1886—Tectonics and Seismicity: U.S. Geological Survey Professional Paper 1313, p. D1–D17.
24. Hamilton, R.M., Behrendt, J.C., and Ackermann, H.D., 1983, Land multichannel seismic- reflection evidence for tectonic features near Charleston, South Carolina, *in* Gohn, G.S., ed., Studies Related to the Charleston, South Carolina, Earthquake of 1886—Tectonics and seismicity: U.S. Geological Survey Professional Paper 1313, p. I1–I18.
25. Heffner, D. M. (2013). *Tectonics of the South Georgia Rift*. University of South Carolina.
26. Herrick, S.M., 1961, Well logs of the Coastal Plain of Georgia: Georgia Geological Survey Bulletin 70, 462 p.
27. King, P. B. (1961). *Systematic pattern of Triassic dikes in the Appalachian region*. *Geological Survey Professional Paper 424-B*. Washington, D.C.
28. Mansfield, W.C., 1937, Some deep wells near the Atlantic coast in Virginia and the Carolinas, U.S. Geological Survey, Prof. Paper 186-I, p. 159-161.
29. Marine, L.W., and Siple, G.E., 1974, Buried Triassic basin in the central Savannah River area, South Carolina and Georgia: Geological Society of America Bulletin, v. 85, p. 311-320.
30. McBride, J.H., 1991, Constraints on the structure and tectonic development of the early Mesozoic South Georgia Rift, southeastern United States; seismic reflection data processing and interpretation: Tectonics, v. 10, p. 1065–1083, doi:10.1029/90TC02682.
31. McFadden, S.S., Hetrick, J.H., Kellam, M.F., Rodenbeck, S.A., and Huddlestun, P.F., 1986, Geologic data of the Gulf Trough area, Georgia: Georgia Geological Survey Information Circular, v. 56, p. 211–214.
32. Milton, C., and Hurst, V.J., 1965, Subsurface "basement" rocks of Georgia: Georgia Geological Survey Bulletin Number 76, 56 p.
33. Neathery, T.L., and Thomas, W.A., 1975, Pre-Mesozoic basement rocks of the Alabama Coastal Plain: Transactions of the Gulf Coast Association of Geological Societies, v. 25, p. 86-97.

34. Nelson, K.D., Arnow, J.A., McBride, J.H., Willemin, J.H., Huang, J., Zheng, L., Oliver, J.E., Brown, L.D., and Kaufman, S., 1985, New COCORP profiling in the southeastern United States. Part I: Late Paleozoic suture and Mesozoic rift basin: Geology, v. 13, p. 714 – 718.
35. Petersen, T.A., Brown, L.D., Cook, F.A., Kaufman, S., and Oliver, J.E., 1984, Structure of the Riddleville basin from COCORP seismic data and implications for reactivation tectonics: Journal of Geology, v. 92, p. 261-271.
36. Pooley, R.N., 1960, Basement configuration and subsurface geology of eastern Georgia and southern South Carolina as determined by seismic-refraction measurements, MS Thesis, University of Wisconsin, 47 p.
37. Ragland, P. A., Hatcher, R. D., & Whittington, D. (1983). Juxtaposed Mesozoic diabase dike sets from the Carolinas: A preliminary assessment. *Geology*, *11*, 394–399.
38. SCDNR, South Carolina Department of Natural Resources Coastal Plain Water Well Records: http://www.dnr.sc.gov/water/hydro/WellRecords/Wells_main.htm
39. Schilt, F.S., Brown, L.D., Oliver, J.E., and Kaufman, S., 1983, Subsurface structure near Charleston, South Carolina – Results of COCORP reflection profiling in the Atlantic Coastal Plain, *in* Gohn, G.S., ed., Studies Related to the Charleston, South Carolina, Earthquake of 1886—Tectonics and seismicity: U.S. Geological Survey Professional Paper 1313, p. H1–H19.
40. Scholle, P.A., 1979, Geological studies of the COST GE-1 well, United States South Atlantic outer continental shelf area: United States Geological Survey Circular 800, 114 p.
41. Smith, D., Review of the tectonic history of the Florida basementMORETESTMORETEST, Tectonophysics, 88, 1-22, 1982.
42. Snipes, D.S., Kidd, N.B., Warner, R.D., Hodges, R.A., Price, V. Jr., and Temples, T.J., 1995, An initial petrographic and geochemical study of a rhyolitic rock recovered from Test Well #1, Hilton Head, South Carolina: South Carolina Geology, v.38, p. 53-60.
43. Steele, K.B., and Colquhoun, D.J., 1985, Subsurface evidence of the Triassic Newark Supergroup in the South Carolina Coastal Plain: South Carolina Geology, v. 28, no. 2, p. 11 – 22.
44. Woollard, G.P., Bonini, W.E., and Meyer, R.P., 1957, A seismic refraction study of the sub-surface geology of the Atlantic Coastal Plain and continental shelf between Virginia and Florida: Madison, University of Wisconsin Geophysics Section, technical report contract no. N7onr-28512, 128 p.
45. Yantis, B.R., Costain, J.K., and Ackermann, H.D., 1983, A reflection seismic study near Charleston, South Carolina, *in* Gohn, G.S., ed., Studies Related to the Charleston, South Carolina, Earthquake of 1886—Tectonics and Seismicity: U.S. Geological Survey Professional Paper 1313, p. G1–G20.
