# Supplementary material for: Limited and localized magmatism in the Central Atlantic Magmatic Province
Source: Nat Commun. 2020 Jul 7;11:3397. doi: 10.1038/s41467-020-17193-6 (PMC7341742; doi:10.1038/s41467-020-17193-6)
Supplement: Supplementary file 3 — Description of Additional Supplementary Files [file 41467_2020_17193_MOESM3_ESM.pdf]

Description of Additional Supplementary Files

File Name: Supplementary Data 1
Description: **Shot, instrument, and pick data for SUGAR Lines 1 and 2**
Tables of shot locations, instrument locations, and pick arrival times used to constrain velocity structure. A detailed summary of all tables is provided in the first worksheet in the file.

File Name: Supplementary Data 2
Description: **SUGAR Line 1 velocity model**
Text file with the velocity model grid for SUGAR Line 1 (Fig. 2a). Data are provided in the following format: distance along line (km, increasing to the NW), model depth (km), velocity (km s^{-1}). Velocities of NaN indicate where the model is not constrained by ray coverage.

File Name: Supplementary Data 3
Description: **SUGAR Line 2 velocity model**
Text file with the velocity model grid for SUGAR Line 2 (Fig. 2b). Data are provided in the following format: distance along line (km, increasing to the SE), model depth (km), velocity (km s^{-1}). Velocities of NaN indicate where the model is not constrained by ray coverage.
